# Ordered and Ushered; the Assembly and Translocation of the Adhesive Type I and P Pili

**DOI:** 10.3390/biology2030841

**Published:** 2013-06-26

**Authors:** James Lillington, Gabriel Waksman

**Affiliations:** Institute of Structural and Molecular Biology, University College London and Birkbeck College, Malet Street, London, WC1E 7HX, UK

**Keywords:** chaperone-usher pathway, type I and P pilus, chaperone, usher, donor-strand complementation, donor-strand exchange, pilicide

## Abstract

Type I and P pili are chaperone-usher pili of uropathogenic *Escherichia coli*, which allow bacteria to adhere to host cell receptors. Pilus formation and secretion are orchestrated by two accessory proteins, a chaperone, which catalyses pilus subunit folding and maintains them in a polymerization-competent state, and an outer membrane-spanning nanomachine, the usher, which choreographs their assembly into a pilus and drives their secretion through the membrane. In this review, recent structures and kinetic studies are combined to examine the mechanism of type I and P pili assembly, as it is currently known. We also investigate how the knowledge of pilus biogenesis mechanisms has been exploited to design selective inhibitors of the process.

## 1. Introduction

Fifty-percent of women will suffer from a urinary tract infection (UTI) during their lifetime. The most prevalent causative agent of UTIs, accounting for 80% of infections, are uropathogenic *Escherichia coli* (UPEC) [1,2]. Upon gaining passage into the host, these Gram-negative bacteria adhere to oligosaccharide-coated receptors in the urinary tract with the aim of forming both intra- and extra-cellular biofilm communities. Their mode of adhesion is via type I (Fim) and P (Pap) pili or fimbriae [3,4]. These are pili formed via the chaperone-usher pathway and are just two of a number of translocated proteinaceous fibres, which are critical to the pathogenesis of some Gram-negative bacteria [5,6]. 

A primarily adhesive role marks chaperone-usher (CU) pili as distinct from structures which act as a tube for substrate transport from bacteria to host (see reviews on type III and type IV (including conjugative pili [7,8]) secretion systems [9,10,11,12]). Other adhesive structures do, however, extrude from Gram-negative bacteria, such as the motility-inducing type IV pili [13,14,15] and curli [16]. Even within the CU pili class, differences may be recorded; one system of categorisation is according to characteristics of the usher protein, producing six clades of α-, β-, γ-, κ-, π- and σ- fimbriae [17]. An alternative categorisation is based upon the length of the loop between the chaperone’s F1 and G1 β-strands: short looped chaperones are classed as FGS (F1-G1-Short) and those with a longer loop as FGL (F1-G1-Long). FGS and FGL systems have distinct architectures: FGL are frequently more flexible with fewer distinct subunits than the FGS. The naming schemes and the diversity that they engender have been extensively reviewed in [18].

The type I or P pili introduced above are γ_1_- and π-clade (respectively) or FGS pili, which mediate host- and tissue-specific adherence to the bladder or the kidney to cause cystitis or pyelonephritis, respectively [19,20,21]. They have been shown to have some non-adhesive roles, in bacterial and host regulatory networks [22,23], but as noted above, their raison d’etre is to bring about the attachment of the bacterial cell to the host. What is fascinating about them is their ability to form without external energy sources via an ingenious mechanism [3] which is now well understood thanks to their remarkable amenability to biochemical and structural studies [24,25]. Not only can any of the proteins involved (including the membrane-embedded usher) be purified and crystallised, but also, they can be made to work *in vitro*, even in detergent. This has allowed us to understand their structure, mode of function and, ultimately, how we might be able to therapeutically inhibit them.

## 2. The Pilus and Its Subunits

**Figure 1:**
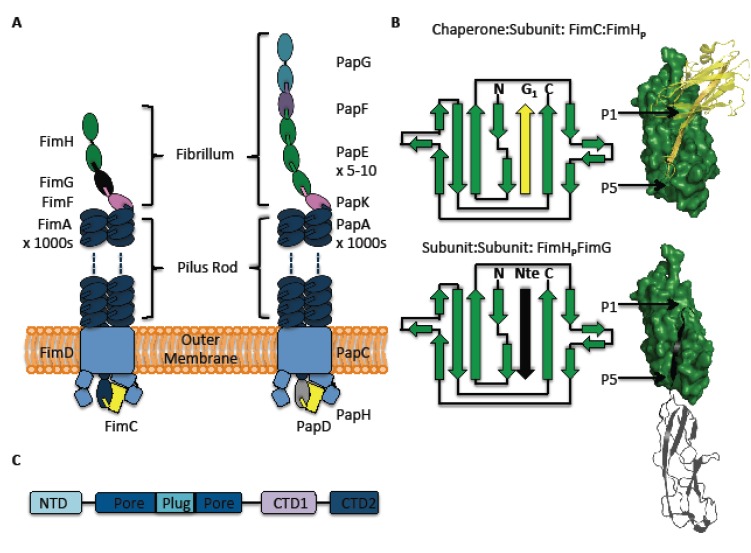
The type 1 and P ushers, pili, donor-strand complementation and exchange. (**A**) Global architecture of the type 1 (left) and P (right) pili. The Fim (type 1) tip (also called “the tip fibrillum”) complex is 10–19 nm in length and comprises the adhesin FimH, followed by one copy of each of the adaptor subunits, FimG and FimF. FimF connects the tip to a rod of FimA subunits, which extend to the outer membrane through the usher, FimD. FimC is the chaperone guiding each subunit to the usher. Similarly, the Pap (P) pilus contains a tip fibrillum complex and a pilus rod. An adhesin (PapG) at the distal end is linked to the rod via tip subunits, PapF (one copy), PapE (5–10 copies) and PapK (one copy). The rod is composed of *circa* 1,000 copies of PapA. The usher and chaperone are PapC and PapD, respectively. In contrast to the Fim system, a terminator subunit, PapH, at the base of the P pilus is identified. (**B**) Topology diagrams and cartoons showing donor-strand complementation in the chaperone:subunit interaction (top) and donor-strand exchange in the subunit:subunit interaction (bottom). The examples given here are FimC:FimH_p_ (coloured yellow:green) and FimH_p_:FimG (coloured green:black), respectively. (**C**) The separate domains of FimD are identified showing the relative positions of the N-terminal domain, pore, plug and C-terminal domains, 1 and 2.

The morphology and subunit architecture of the type I and P pili have been examined for over 20 years [26,27,28], resulting in a fine level of detail now being understood (see reviews [3,5,29,30] ). Each pilus is a polymer of pilus subunits or pilins, passing through the bacterial outer membrane and protruding approximately 2 µm into the extracellular milieu (Figure 1A). It is anchored to the membrane by the usher—FimD and PapC for type I and P pili, respectively—an outer membrane protein used for assembly and secretion of pilus subunits. The pilus itself is composed of two sections: the rod and the tip fibrillum (Figure 1A). The tip fibrillum contains only three Fim proteins in type 1 pili (one copy each of FimH, FimG and FimF), but about 13–18 Pap proteins in the P pilus (one copy of PapG, one copy of PapF, 5–10 copies of PapE and one copy of PapK). FimH and PapG are the ‘business end’ of the pilus, as these “adhesins” mediate recognition of and binding to host-cell receptors. The right-handed helical rod (3.3 subunits per turn) makes up most of the 2 µm pilus length, being comprised of thousands of copies of the major pilin, FimA/PapA [28,31]. Its external diameter of 7–8 nm is in stark contrast to the much more slender FGL pili (2 nm diameter), of which the F1 capsule of *Yersinia Pestis* is the archetype. F1 capsules are also tip-less, being polymers of a sole subunit, Caf1 (see reviews [5,32]). Full details of the type 1 and P pilus subunit nomenclature and ordering are given in the legend of Figure 1. Of note is a terminator subunit found in the P pilus (PapH), which abrogates polymerization and, as result, is always found at the base of the P pilus [33]. No terminator equivalent has been found in the Fim system; a comparison of gene architecture between P and type I pili had led to FimI being proposed as the PapH homologue [34]; however, the phenotypic evidence for this is lacking [35].

Prior to pilus formation, individual pilus subunits or pilins are exported through the inner membrane and to the periplasm by the Sec pathway [36] and guided to the usher via a chaperone, FimC/PapD, in the type 1 and P pili systems. The chaperone is crucial in promoting and catalysing pilin folding [37,38] and also provides a checking function—only correctly disulfide bonded moieties may be bound and, hence, transported to the pilus [39]. Pilins are single domain, incomplete immunoglobulin (Ig) -like structures—except for the adhesins, FimH and PapG, which additionally contain an extra lectin domain for their adhesin function. For the FimH proteins, these domains shall be referred to in the text as FimH_P_ (for pilin) and FimH_L_ (for lectin).

Each pilin’s Ig structure is incomplete, since they lack a seventh C-terminal β-strand, strand G, leaving a gaping hydrophobic groove on the pilin’s surface (Figure 1B). This unstable arrangement is rectified by insertion of the G1 strand of the boomerang-shaped chaperone, so burying the hydrophobic groove of the pilin (Figure 1B) [40,41]. This interaction is termed ‘donor-strand complementation’, as the chaperone donates one of its own strands to complete the subunit’s fold. It enables the chaperone to stabilise the subunit and prevents any premature interactions between subunits. The key interactions in the highly conserved chaperone:subunit interface are between four alternating hydrophobic chaperone residues (P1-4 residues) and four of the five prominent hydrophobic patches in the subunit groove (termed P1-P5 pockets) [40,41,42]. However, the chaperone β-strand inserts unusually within the groove of the subunit domain: it inserts parallel to strand F, a higher energy conformation than the one found usually in Ig-folded structures, where strand G inserts antiparallel to strand F.

In addition to an incomplete Ig-like fold, each pilin (except FimH/PapG) also has an N-terminal extension (Nte) of 10–20 residues. Similarly to the chaperone G1 strand, these Ntes contain five alternating hydrophobic residues (also termed P1–P5 residues). During pilus biogenesis, an incoming subunit will use its Nte to wrestle the G1 chaperone strand out from the preceding subunit groove, so releasing the chaperone and becoming the next subunit in the chain (Figure 1B). This process is called ‘donor-strand exchange’ (DSE).

In more detail, initial attack by the incoming subunit Nte is upon the unoccupied P5 pocket of the preceding subunit groove. This attack is carried out by the P5 residue of the incoming subunit Nte, which inserts into the P5 pocket of the preceding subunit groove. This positions the Nte at the entrance of the preceding subunit’s groove for invasion of that groove. From here, the incumbent chaperone P1-4 residues are displaced one by one from their position in the preceding subunit cleft in a process resembling a zip-in/zip-out mechanism [43,44,45]. Finally, having displaced the chaperone, the pockets P2-5 lie occupied, and the donated Nte lies anti-parallel to strand F in the Ig-domain of the subunit, resulting in the more energetically favourable arrangement [46]. The interaction surface is highly conserved—an example is the sterically constraining Pap P4 site, which may only accept an incoming glycine residue from any of the Ntes. However, subtle differences, particularly (but not exclusively), which amino acids may be accepted by the P5 pocket, help define which subunits are neighbours or ‘cognate pairs’ [42,47]. The ordering of subunit incorporation into the pilus is reviewed below.

Using donor-strand exchange, the growth of the pilus, therefore, continues; subunits from incoming chaperone-subunit complexes are added to the base of the nascent pilus one at a time and the chaperone recycled. The Nte-groove interaction between subunits is extremely strong (one of the strongest non-covalent interactions known in nature) and is much stronger than the chaperone:subunit interaction, explaining why the DSE reaction is energetically driven towards pilus subunit polymerisation [3,46,48]. It also helps to impart great mechanical strength upon the extendable pilus, allowing the shear forces encountered by the adhering bacteria to be weathered [49]. 

## 3. How the Subunits are Processed by the Usher

### 3.1. Mechanism of Subunit Polymerization at the Usher

The usher is a 100 kDa outer-membrane-spanning protein comprised of five domains [25]: a periplasmic N-terminal domain (NTD), two C-terminal domains (CTD1, CTD2 respectively), a plug domain, as well as an outer membrane spanning pore (Figure 1C). Whilst the chaperone is required to prevent premature subunit interactions, the usher is equally important in ensuring that, at the time and place required, the subunits do interact productively. It does this through the concerted involvement of its periplasmic domains (NTD, CTD1, CTD2 and plug), with an usher monomer being sufficient for the entire process, despite some evidence of dimerization [50,51]. 

In the type 1 pilus system, pilus biogenesis starts with the recruitment of the FimC:FimH complex to the usher NTD. Although the NTD is the primary recruitment site for all chaperone:subunit complexes, its affinity is highest for the chaperone:adhesin complex (FimC:FimH and PapD:PapG in the type 1 and P pilus systems, respectively) [52,53,54,55]. A ‘two prolines lock’ allosteric mechanism ensures that only chaperone:subunit complexes, and not free chaperone, bind this NTD [56]. By an unknown mechanism, the usher plug, which, in the resting state, has been maintaining the integrity of the membrane by occluding the usher pore, moves to a new position near the NTD. Here, it appears to play an important role in the recruitment of future subunits [55]. 

**Figure 2:**
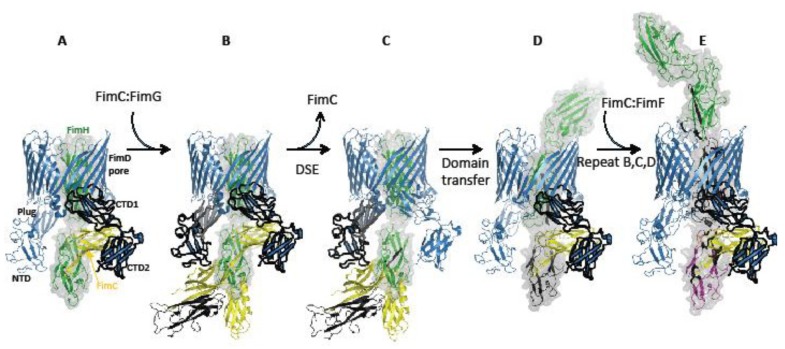
Formation of the type 1 tip complex: (**A**) The FimD:FimC:FimH complex (PDB 3RFZ). All usher domains are labelled, as well as FimC and FimH. (**B**) Modelled addition of FimC:FimG to FimD:FimC:FimH prior to DSE. FimC:FimG binds to the N-terminal domain (NTD) of FimD with its disordered Nte oriented towards the P5 pocket of FimH in preparation for donor-strand exchange (DSE) [25]. (**C**) FimG undergoes DSE with FimH, displacing FimC from the FimC:FimH complex and removing it from the FimD C-terminal domains (CTDs) in preparation for translocation. (**D**) FimC:FimG transfers to the CTDs. In doing so, FimH_L_ is translocated inside the FimD pore. (**E**) After a repeat of steps B, C and D with FimF instead of FimG, the tip assembly complex is complete, with FimH fully translocated (PDB 4J3O) [59]. FimD is coloured in blue, FimC in yellow, FimH in green, FimG in black and FimF in magenta. Domains of FimD, which interact with the chaperone/subunits in any given step, are outlined in black.

After being targeted initially to the NTD, FimC:FimH relocates to the FimD CTD domains during translocation [25,57]. Binding competition experiments [55] and the observation of quaternary complexes comprising NTD-CTD-chaperone:subunit moieties [58] suggests that it is an allosteric handover, rather than the competitive procuring of the same binding site. This passing from NTD to CTDs frees up the NTD and plug region for binding of the next incoming chaperone:subunit complex, FimC:FimG. Binding of FimC:FimG to the NTD positions the Nte of FimG directly next to the FimH_P_ groove, and thus, DSE occurs immediately between the two subunits, leading to the removal of the FimH-bound chaperone from the CTDs. In the next step, FimC:FimG transfers to the CTDs, freeing the NTD for recruitment of the next chaperone:subunit complex in assembly, FimC:FimF, and the whole cycle repeats again to not only complete the tip complex, as shown in the recent FimD:FimC:FimF:FimG:FimH crystal structure, but also the entire FimA rod [59]. This mechanism is shown in Figure 2. 

### 3.2. Regulation of Ordering

Correct ordering of subunits ensures that a functional pilus will result [60,61]. For the type 1 pili (and also likely for the P pili), involvement of the usher is a strict requirement to catalyse donor-strand exchange, enhancing the rate significantly [62]. A reasonable question to ask, therefore, is whether subunit ordering is due to the usher or simply due to the innate properties of the subunits themselves. The answer appears to be that both of these options play a role.

In both the Fim and Pap systems, the usher NTD binds the chaperone:adhesin complex more strongly than it does any other chaperone:subunit complex—K_d_ 0.9 mM for FimD interaction with FimC:FimH [52,53,55,63] and 3.2 nM for PapC interaction with PapD:PapG [55]. Volkan *et al.* were unable to detect any interaction between the NTD of PapC and the other chaperone:subunit complexes (except PapD:PapE) [55], although interactions were recorded in the gas phase by Morissey *et al.* [58]. This strong chaperone:adhesin binding relative to other subunits may help ensure that it always initiates the proceedings. For the Pap system, the usher’s plug domain appears to be important in aiding the NTD in its recruitment of the subsequent subunits, providing an interesting use for a domain that had appeared to have fulfilled its purpose prior to pilus growth, *i.e*., blocking the usher pore in its resting state [55]. In contrast, the FimD NTD does observably bind its chaperone:subunit complexes, FimC:FimG, FimC:FimF and FimC:FimA. However, the affinities that are observed are not aligned with the order in which subunits are found in the pilus, being 27 mM for FimC:FimG, 6.6 mM for FimC:FimF and 29 mM for FimC:FimA. From these values, one might expect that FimC:FimF would undergo DSE with FimH. This event has actually been observed, although far less frequently than it should be solely on the basis of these affinities [28,63]. 

Since the rank order of chaperone:subunit complex affinities for the usher NTD does not reflect the order in which subunits are found in the pilus, then, perhaps, subunit order regulation comes from different DSE rates between pairs of subunits. That is, a subunit recruited earlier in the process would need to have a faster DSE rate compared to a subunit with stronger binding affinity for the usher, but recruited later in the process. Despite the sequence similarity (*ca*. 65% for the Fim subunits [59]), it has since been shown that there is indeed DSE rate differentiation. A full quantitative picture of the rates of usher-mediated donor-strand exchange in the type I pilus is now available [51]. It shows that the rates of DSE follow the expected trend for all the cognate pairings: FimG undergoes the fastest DSE with FimH (171 min^−1^), followed by FimF with FimG (3 min^−1^), then FimA with FimF (0.03 min^−1^). Non-cognate subunit pairs react with each other much more slowly than any given cognate pairs, and with the exception of the subunits found in multiple copies in functional pili (FimA, PapA and PapE), subunits undergo only very slow DSE with themselves [51,64]. 

Interestingly, once the tip (FimH, FimG and FimF) complex is in position and completed, the FimA-FimA DSE is shown to be the fastest process of any (960 min^−1^), polymerising over 1,000 copies into the dense rod formation [62]. The low rate of DSE by FimA with FimF is perhaps to be expected: given the high rate of FimA self-association and the presumable high concentration of it in the periplasm required to provide the pilus scaffold, an incorrect or early incorporation of FimA would rapidly lead to the waste of many FimA subunits. 

It appears, therefore, that DSE rates are the greater determinants of the subunit’s final positioning in the pilus. This is supported by DSE data in absence of the usher, showing that when Nte peptides are reacted with chaperone:subunit complexes, despite being much slower than when catalysed by an usher, the ordering of DSE rate is unchanged [65]. The cause of rate differences is to be found in the chemical makeup of the Nte, notably, the nature of the residue at the P5 position and immediately surrounding it (P5+1 and P5−1): thus, the interaction between the incoming subunit Nte’s P5 residue with the preceding subunit’s P5 pocket appears to be the largest rate-influencing factor [43,47,65]. Since the DSE reaction does not occur in isolation, however, the role of the usher, as well as the relative concentrations of subunits in the periplasm, should not be disregarded—particularly in the major events of initiation and termination. It is only when accounting for each of these that the wild type observations may be explained [51]. 

In the P pilus system, termination of pilus biogenesis is caused by the recruitment of the terminator subunit, PapH. Once bound to the usher NTD, PapH undergoes DSE with the PapA subunit, which has been added in the previous cycle of subunit incorporation, but subsequent chaperone:subunit recruitments and DSE reactions are prevented. There are two reasons for this: (i) PapH has no P5 pocket in which to receive a subunit Nte’s P5 residue [33], and thus, PapH is prevented from undergoing DSE with another subunit; and (ii) the PapD:PapH has no affinity for the usher CTD2 domain and, so, might be unable to transfer to the CTDs, thus preventing recruitment of additional subunits to the usher NTD [55]. 

## 4. Passing of Subunits through the Usher

**Figure 3:**
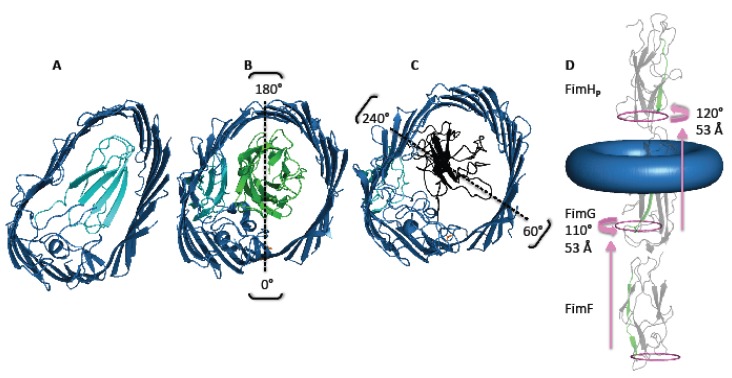
Shape and contacts of the FimD usher. **(A)** The pore (dark blue) displays an oval-shape and is filled by the plug domain (cyan) prior to secretion (PDB 3OHN). **(B)** Insertion of FimH (green) into the pore leads to removal of the plug and a change in pore shape to an inner circular tube (PDB 3RFZ) [25]. The strongest contacts between the pore and FimH are around 0° and 180°, taking FimD Asp208 (coloured in orange) as the 0° reference point. **(C)** In contrast, the strongest contacts when FimG (black) resides in the pore are around 60° and 240° (PDB 4J3O) [59]. **(D)** Subunits rotate by 110–120° in an anticlockwise direction during translocation. Translation distances per subunit through the FimD pore is indicated by pink arrows. The start and end orientations for a homologous β-strand from FimF, FimG and FimH_p_ are shown in green (FimF A133-N123, FimG A113-T123, FimH V248-R258).

Difficulties intrinsic to the handling of transmembrane proteins have, for a while, slowed down research on the usher itself. While the events taking place in the periplasm (DSC, DSE and usher domain interaction with chaperone:subunit complexes) were starting to emerge, events occurring at the membrane were less understood. This situation changed dramatically in 2008, starting with the original PapC translocation domain crystal structures, which provided fascinating insights into the largest transmembrane β-barrel ever seen [66,67]. These structures highlighted the unusual nature of a plug, which rather than being found at the N-terminus of the barrel, was actually contained within it. An α-helix and a β5-6 hairpin were thought to support the plug and expected to provide an energetic release for plug displacement and subunit translocation [66,68] (Figure 3A). These features were also observed in the structure of the FimD translocation domain [25]. 

It was a surprise, therefore, to find upon determination of the FimD:FimC:FimH structure that after rehousing of the plug, these features remained largely unchanged [25]. What had changed, however, was the global architecture of the pore. The FimD barrel reformed upon engagement with FimC:FimH from an oval to a circular shape (Figure 3A-B) and had increased its cross sectional area by *ca*. 100 A^2^ [25]. The solution of the complete tip complex FimD:FimC:FimF:FimG:FimH structure and the accompanying energetic studies therein [59] have shown just how individually accommodating the pore is. Rather than simply providing generic passage through the membrane, the FimD pore lumen contains residues that are specific and distinct for each moiety that passes through it. The strongest interaction surfaces between any subunit and pore are arranged diametrically opposite of each other, so that the subunit is kept in the very middle during translocation. Furthermore, comparing specific subunits, the strongest interactions between the FimH_L_ domain and the pore are at approximately 90° to the strongest interactions between FimG and the pore (Figure 3B-C). 

These different interactions are reflected by the differences in translocation energy for each subunit. For example, there is a low translocation energy barrier for FimG to move through the barrel, but a high one for the translocation of FimH_L_. Since all subunits/domains (except FimH_L_, which has a jelly-roll fold) are structurally homologous to FimG (they all form a pilin domain), it has been suggested that translocation of all pilus subunits (except the adhesin) is energetically cheap. Interestingly, the energy barrier to take the plug domain out of the usher pore is also low. Previous electrophysiology measurements have shown fleeting ‘off-guard’ movements of the PapC plug, allowing short periods of high apo-usher conductance. Taken together, the dynamism of the plug and the strength of the FimH-barrel interaction relative to that of the plug-barrel interaction would provide the opportunity for a competitive displacement mechanism of the plug by FimH [59,68]. However, the discovery of plug binding to the PapD:PapG_P_ (PapG_P_ being the pilin domain of the PapG adhesin) complex prevents an allosteric mechanism from being ruled out [55].

Translocation might be energetically cheap, but is still associated with a cost, however small this cost might be. The molecular basis of translocation is still unknown, but it has been suggested that translocation might be facilitated by conformational changes occurring within the nascent pilus as it emerges from the usher pore. A conformational change has been observed within FimH: in the FimD:FimC:FimH structure, the pilin and lectin domains of FimH are aligned, while in the FimD:FimC:FimF:FimG:FimH structure, the two domains have closed in. In the first structure FimH_L_ is inside the pore, and FimH_P_ is bound to the CTDs on the periplasmic side of the structure, while in the second structure, both domains have emerged from the pore and are both on the extracellular side of the pore. Thus, the two structures provide a snapshot of FimH pre- and post-transport. It has been suggested that the conformational change occurring post-transport prevents FimH from back sliding within the pore and, thus, would contribute to unidirectional translocation of FimH. Other conformational changes, affecting the major FimA/PapA subunits, are also thought to prevent backtracking of pilus subunits through the pore: indeed, both FimA and PapA emerge from the pore to adopt a super-helical arrangement of 3.3 subunits per turn in the rod. This results in a protein polymer super-helix of 70Å in diameter [28], which cannot insert back within the usher pore. Super-helix formation could also exert a pulling force on the pilus to extract it out of the pore if the energy released in the process were to be superior to the energy needed to destabilise subunits interactions within the pore lumen. Given the stability of the super-helix and the weak forces maintaining subunits, like FimG, within the pore, this mechanism appears at least plausible.

Another energetic barrier, which must be overcome during the subunit incorporation cycle, is the transfer of chaperone:subunit complexes from the usher NTD, where they are initially recruited, to the CTDs, where they transfer after DSE has occurred. Evidence has been provided suggesting that release from the usher NTD is allosterically driven by the usher CTD2 domain [55]. Indeed, CTD2 alone is able to destabilise the NTD interaction with chaperone:subunit complexes. DSE of the NTD-bound subunit with the CTD-bound one might also destabilise NTD-chaperone:subunit interactions, although this remains to be shown. More recently, the FimD:FimC:FimF:FimG:FimH structure has unravelled an additional and likely important actor in the NTD-to-CTD transfer mechanism [57]. In this study, an energy path along the pore lumen was discovered, which imposes on the subunits a combined translation/rotation of about 53Å/110–120° (Figure 3D). Thus, as the pilus is pulled out on the extracellular side by the combined action of CTD2-mediated destabilisation of NTD-chaperone:subunit interaction and the formation of the rod, a translational/rotational motion is imposed on the nascent pilus, leading to the relocation of the chaperone:subunit complex at the base of the pilus from the NTD to the CTDs.

## 5. Emergence from the Pore and Adhesion

Upon translocation to the external milieu, the pilus must fulfil its adhesive function. The Fim and Pap pili are examples of monoadhesive pili, with adhesion being mediated entirely by the terminal adhesin subunit. Other pili (particularly, the FGL pili) are polyadhesive, where each protein subunit is able to adhere to the host; binding sites exist, therefore, along the exposed surface of the pilus [69,70,71]. 

**Figure 4:**
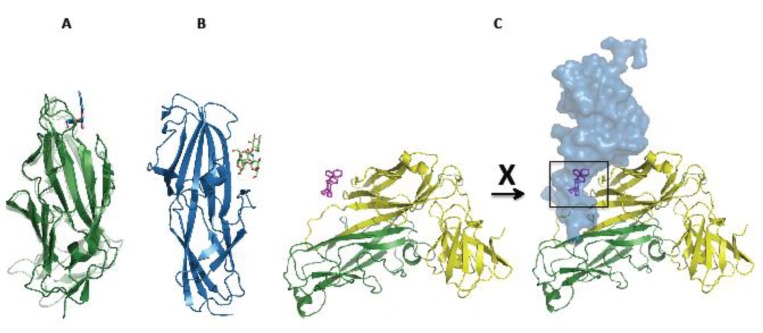
Adhesin and pilicide binding sites. **(A)** FimH_L_ binds a mannose moiety via a distal pocket. In so doing, the pocket tightens around it. Pre-mannose binding FimH_L_ is coloured light green (PDB 3JWN [78]), mannose-bound FimH_L_ in dark green and mannose in blue (PDB 1UWF) [99]. The mannose binding event also leads to structural changes at the opposite end of FimH_L_, the region in contact with FimH_P_ [78]. **(B)** PapG adhesin displays side-on binding of the globoside. The PapG lectin domain is coloured in blue and the GbO_4_ globoside in green (PDB 1J8R) [75]. **(C)** Current pilicide design targets a hydrophobic chaperone patch, which would normally bind to the usher NTD, so preventing the interaction with the usher. The structure of PapD:PapG_L_ bound to the pilicide ‘2C’ (coloured in yellow, green and magenta, respectively, PDB 2XG4) is incompatible with concurrent binding to the usher NTD (blue surface, FimD taken in homology to PapC by alignment with PDB 1ZE3 [100]), due to clashing of pilicide and usher, as has been verified experimentally [90,101].

The FimH adhesin binds the ubiquitous D-mannosylated receptors, such as uroplakins, mainly, but not exclusively, to those of the bladder epithelium [18,72,73]. The PapG adhesin targets the Galα-1,4 Galβ receptors present on the kidney epithelium—the particular glycolipid bound depends on which of the three PapG isoforms is being challenged [74,75]. Despite the homologous nature of these two pili, the mechanisms of binding appear quite distinct, with FimH binding via a deep binding pocket at its tip [41], whereas PapG has a more shallow, side-on arrangement (Figure 4 A-B) [75], posing questions as to the different orientation of the bacterial approach for attack by each pilus [75]. The different specificities of these pili explain the apparent redundancy of pilus production between the type I and P pili of UPEC. A synergic role has been suggested between them [19], with the pursuit of different targets maximising the bacterial adherence safety net [76]. 

Even when binding the correct oligosaccharide substrate, the FimH adhesin exhibits interesting binding properties: binding affinities differ depending on whether its substrate is free in solution or anchored to a solid surface. FimH indeed exhibits a ‘catch bond’ binding recognition mode; that is, a strength of interaction, which increases with the amount of force exerted on it [77]. Whilst the actual interaction is at the tip, it is the connectivity between the two FimH domains that determines how tightly the mannose is bound. When they are stretched, the mannose binding pocket at the distal end clamps shut—a mechanism likened to a ‘finger trap’ toy (Figure 4A) [78]. This work has been corroborated by the FimD:FimC:FimF:FimG:FimH structure and molecular dynamics simulations on the fimbrial tip, which show that upon translocation from the usher, FimH emerges in the relaxed state as part of a flexible tip, with domains closing in and the mannose binding pocket opening, ready for the receipt of a receptor [59,79].

The FimH-mannose interaction can therefore be in a low affinity or a high affinity state, capable of withstanding <20 pN or 30–70 pN, respectively, depending on the stress it is put under. The hypothesis for this catch bond mechanism is that the bacteria may remain adhered to mannosylated surfaces during stress, e.g., urinary washing or targeting by the immune system [80], but in the event of binding to solubilised mannose moieties—useless for adhering to—they will quickly dissociate. Supporting evidence for this comes from the emergence of naturally occurring FimH mutations, which bind mono-mannose more tightly, but with the loss of the catch bond ability [81]. The occurrence of these strains and other variability in FimH [82] shows their viability in some environments; however, they appear less successful in arenas where the bacteria is subjected to high urinary-flow stress, such as in the urinary tract [83].

Pili also have key roles, other than their adhesion function, in pathogenesis, For example, type I pili have a role in triggering actin rearrangement for enabling bacterial uptake [84] and in maintenance of both extra- [85] and intra-cellular [84,86] biofilm communities. Indeed, FimH residues, which are unrelated to the adhesin role, are critical for this function [83]. The next section details efforts to develop antimicrobials targeting pilus adhesion; however, the non-adhesive properties may prove just as fruitful for therapeutic exploitation, since bacteria unable to enjoy the comfort of a biofilm are more exposed to the brunt of the host immune system.

## 6. Preventing Adhesion

The increasing knowledge of the function of pili, combined with the annual large cost of UTIs and the decreasing effectiveness of antibiotics, has generated interest in the therapeutic targeting of pili. A drug that combats a non-essential bacterial function, as opposed to an antibiotic, is thought to induce a reduced rate of bacterial resistance towards it [87]. However, the production of a silver bullet generic for pili appears hampered by the relatively low binding power of some of the adhesin sites, with adhesion seemingly dependent on a ‘strength in numbers’, as well as clever bacterial avoidance of free flowing competitive inhibitors by the catch bond mechanism [88]. 

Nevertheless, a number of strategies have been followed in the pursuit of type 1 and P pilus targeting drugs. These target (i) the usher interaction with the chaperone-subunit complex (ii) adhesin-receptor binding and (iii) ability of the bound pilus to weather forces exerted upon it [89] (see [88] for an extensive recent review).

A series of bicyclic 2-pyridones, termed pilicides, were developed with interaction (i) in mind [90]. These bind the chaperone at the back of the F1-G1 loop region, an area that interacts with the usher NTD (Figure 4C). As such, they are effective in blocking the interaction between usher and the chaperone:subunit complexes and, hence, pilus formation, leading to a reduction in adherence and biofilm formation [90].

Prevention of FimH adhesion is the goal of the ‘mannosides’, disruptors of interaction (ii). These have been designed to mimic the mannose-like properties of the FimH binding partners. Competitive binding and, hence, abrogation of adhesion are reported to inhibit colonization and formation of intracellular bacterial communities [91]. The first murine models for the effectiveness of mannosides are now being reported [92,93]. Parallel studies for the diminution of binding in the Pap system have also been conducted [94,95], including one using a bacterial coating receptor mimic for PapG [96]. Happily, Pap adhesion was found in a further study to be independent of pH, avoiding the necessity to significantly alter the urinal pH of patients [97]. 

Finally, ‘coilicides’ aim to modulate the ability of pili to weather shear forces by urinary flows (see (iii) above). The ability of the pilus super-helical rod to uncoil and re-coil depending on forces applied to it is a powerful means by which groups of pili can act in concert, so reducing the chance of catastrophic (to the bacteria) breakage. When purified PapD is introduced to a pilus to which forces have been applied (by optical tweezers, for instance), it decreases the ability of the pilus to undergo multiple uncoiling and recoiling cycles. PapD probably interacts with PapA subunits in the stretched pilus, impeding the reformation of interlayer interactions [98]. This leads to a new concept in the search for novel drug candidates targeting subunits, which compose the pilus rod.

## 7. Concluding Remarks

The type I and P pili have proven themselves amenable to purification, manipulation and study. As a result, the major aspects of pilus biogenesis by the chaperone:usher pathway are largely understood. We know that during pilus biogenesis, pilus subunits are added sequentially one at a time in a subunit incorporation cycle mechanism for which unprecedented levels of detail have been obtained. Outstanding questions remain, however. Firstly, little is known of the conformational changes taking place during usher activation by the FimC:FimH complex. The structure of a full-length usher in its apo form would be useful to elucidate some of the steps leading to domain rearrangement within the usher. The only apo structure available for the usher is of its translocation domain: thus, we know the plug domain moves out of the pore to let in FimH_L_, but what happens to the usher NTD or CTDs? Do they in any fashion assist in pore extrusion or FimH_L_ insertion? These questions remain to be answered. Similarly, a number of steps in the NTD-to-CTDs transfer of chaperone:subunit complexes remain to be documented: for example, one question remains as to how exactly chaperone:subunit complexes are released from the NTD after DSE. Finally, the molecular basis for translocation is unclear: is rod formation an important player in this process? These questions will no doubt keep the field vibrant in the years to come. We also anticipate that drug design efforts targeting pilus biogenesis will continue unabated. Given the increased damage UTIs cause in hospitals, there is a need for new therapeutic interventions, particularly at the catheterisation stage, where most UTIs emanate.
